# Nocebo effects in long-term health conditions: a systematic review of experimental studies

**DOI:** 10.3389/fpsyt.2026.1752434

**Published:** 2026-05-08

**Authors:** Billy A. Church, Pankaj Gupta, Adarsh Shivaram, Sian Jenkins, Emma J. Palmer

**Affiliations:** 1 School of Psychology and Vision Sciences, University of Leicester, Leicester, United Kingdom; 2 Department of Cardiovascular Sciences, University of Leicester, Leicester, United Kingdom; 3 Leicester British Heart Foundation Centre of Research Excellence, Leicester, United Kingdom; 4 Leicestershire, Northamptonshire and Rutland (LNR) GP Training Programme, NHS England, Leicester, United Kingdom

**Keywords:** adverse events, experimental studies, long-term conditions, negative expectations, nocebo effect, patient communication, symptom perception, systematic review

## Abstract

**Background:**

The nocebo effect refers to adverse symptoms triggered by negative expectations rather than active treatment mechanisms. These responses can undermine treatment adherence, increase symptom burden, and reduce clinical effectiveness. Patients with long-term conditions (LTCs) may be particularly vulnerable because they frequently interact with healthcare systems, monitor symptoms closely, and often have prior negative treatment experiences. Despite these important clinical implications, experimental evidence on nocebo responses in LTC populations has not been systematically synthesised. This review evaluated induction methods, symptom domains, and potential moderators to inform strategies for reducing avoidable nocebo-related harm in clinical practice.

**Methods:**

The review followed PRISMA 2020 guidelines and was preregistered on OSF. MEDLINE, Web of Science, PsycINFO, and CINAHL were searched to 21 August 2025. Eligible studies experimentally induced nocebo effects in adults with physical or neurological LTCs using verbal suggestion, conditioning, or negatively framed communication. Quality was assessed using the Mixed Methods Appraisal Tool (MMAT). Due to substantial heterogeneity in induction procedures and outcome measures, findings were synthesised narratively.

**Results:**

Thirteen studies met the inclusion criteria, covering chronic pain, asthma, Parkinson’s disease, cancer, and dermatology. Nocebo effects were commonly induced through negative verbal suggestion or negatively framed clinical information. Several studies demonstrated increased subjective symptom intensity, alterations in physiological markers, and brain activation patterns associated with expectancy. Evidence was mixed for neurological symptoms. Anxiety and other psychological moderators were measured inconsistently, and outcome definitions varied considerably across studies.

**Conclusion:**

Individuals with long-term conditions can show strong nocebo responses across a range of symptoms. These findings highlight the importance of how treatment information is communicated in clinical encounters. More consistent induction and measurement approaches, routine assessment of psychological moderators, and evidence-based communication strategies are needed to reduce unnecessary symptom burden and improve treatment engagement in long-term condition management.

**Systematic review registration:**

Open Science Framework, https://osf.io/nj7yt/.

## 1 Introduction

The nocebo effect is a phenomenon observed across many areas of healthcare. Originally defined as negative placebo responses (1), the term is now commonly used to describe adverse outcomes arising from inert or non-pharmacological factors (2–7).

The nocebo effect describes the experience of adverse outcomes that result not from an active treatment agent, but from a patient’s negative expectations regarding treatment. These effects, though psychologically or contextually mediated, can produce real and clinically significant symptoms such as pain, nausea, itch, and fatigue. Nocebo effects can reduce treatment efficacy, compromise adherence, increase side-effect reporting, and undermine patient-clinician trust (8, 9).

In line with contemporary consensus, it is important to distinguish between nocebo responses and nocebo effects (10). Nocebo responses refer to the observed adverse symptoms or outcome changes following an inert or contextual manipulation, whereas nocebo effects denote the component of these responses that can be causally attributed to psychosocial mechanisms such as expectations, learning, or contextual cues, beyond natural disease progression or other non-specific factors. Throughout this review, the term nocebo effect is used specifically to refer to experimentally induced adverse changes inferred to arise from these psychosocial mechanisms.

Compared to placebo responses, nocebo phenomena have received relatively limited research attention, despite their growing relevance in clinical trials and routine care. In real-world settings, patients often discontinue effective medications due to symptoms they believe are caused by the treatment; even when those symptoms are nonspecific or unrelated (11). These reactions may be amplified by prior negative experiences, verbal suggestions, warnings in medication leaflets, or anxiety surrounding side effects. Clinicians may inadvertently contribute to nocebo responses by overemphasising potential adverse events or failing to provide context when discussing treatment risks (12).

Nocebo responses may be particularly pronounced in individuals with long-term conditions (LTCs), such as chronic pain, neurological disorders, and respiratory disease. These individuals typically engage with healthcare systems more frequently, may have a heightened awareness of bodily symptoms, and are more likely to have encountered previous adverse treatment experiences. Chronic illness also involves sustained symptom monitoring, which may increase the likelihood that naturally occurring or ambiguous symptoms are misattributed to treatments. For instance, among patients prescribed statins for cardiovascular risk, muscle pain is one of the most frequently cited reasons for discontinuation. Yet controlled trials have suggested that up to 78% of reported muscle symptoms may stem from the nocebo effect rather than the medication itself (13).

Nocebo responses are not purely subjective. Studies have shown measurable changes in brain activity, immune responses, and hormonal levels following expectancy manipulation. For example, cortisol levels have been observed to rise following negative verbal suggestions about treatment outcomes, and functional neuroimaging has revealed activation in pain- and anticipation-related brain regions during induced nocebo states (8). These physiological effects lend weight to the notion that the nocebo effect is not merely a reporting bias or artefact but a genuine biopsychosocial phenomenon.

To date, several systematic reviews have been published that examine the nocebo effect. Some have focused on gender differences (14), non-specific adverse effects (15), pain (2), the neurobiology of nocebo responses (7), and the role of anxiety (16). Broader syntheses, such as umbrella reviews, have also been conducted (17, 18). However, no systematic review has comprehensively examined the nocebo effect across people with long-term conditions. Instead, systematic reviews have focused on specific drug classes or conditions, such as fibromyalgia (19), migraines (20), and antidepressant medications (21).

Moreover, there is considerable methodological heterogeneity in the way nocebo effects are studied. Some experiments use verbal suggestion alone, while others combine negative framing with conditioning paradigms. Outcome measures also vary widely, from self-reported symptom scales to physiological indices such as cortisol, respiratory flow, or neuroimaging signals. This variation makes it difficult to draw generalisable conclusions about the reliability and clinical significance of induced nocebo responses.

Given the increased global emphasis on patient-centred care, shared decision-making, and informed consent, understanding the mechanisms and consequences of the nocebo effect has practical implications. Poorly framed communication may not only increase side-effect reporting but may also erode patient engagement with effective therapies. Conversely, identifying the contexts and populations most at risk of nocebo effects may allow clinicians to tailor their communication strategies and reduce unnecessary treatment discontinuation.

### 1.1 Objectives

The aim of this systematic review was to identify and synthesise experimental evidence of induced nocebo effects in individuals with physical or neurological long-term conditions. Specifically, we sought to answer the following research questions:

- What magnitudes of effect have been reported for experimentally induced nocebo responses in LTC populations?
- Do these effects vary by age, gender, or psychological traits such as anxiety?
- What methods and tools are used to induce and measure nocebo responses in these studies?
- Which long-term conditions have been studied, and where are the gaps?

The review was conducted in accordance with the Preferred Reporting Items for Systematic Reviews and Meta-Analysis (PRISMA 2020) guidelines, and all procedures followed open science best practices, including prospective protocol registration, transparent reporting, and plans for data sharing (22).

## 2 Methods

### 2.1 Protocol and registration

This systematic review was conducted in accordance with the Preferred Reporting Items for Systematic Reviews and Meta-Analyses (PRISMA) 2020 guidelines (PRISMA 2020). The review protocol was prospectively registered on the Open Science Framework (OSF) (https://osf.io/nj7yt/overview).

### 2.2 Eligibility criteria

Studies were eligible for inclusion if they met the following criteria:

1. employed an experimental design involving an inert or non-pharmacological manipulation framed within a nocebo context;
2. included adult participants with a diagnosed long-term health condition;
3. examined adverse outcomes, including symptom change, side-effect reporting, or experimentally evoked symptoms;
4. were published in a peer-reviewed journal in English.

Consistent with contemporary conceptual frameworks, nocebo effects were defined as adverse changes in outcomes that could be causally attributed to psychosocial mechanisms, such as expectancy, contextual cues, or learning, rather than reflecting background symptom fluctuation, disease progression, or other non-specific factors (10).

Accordingly, studies were considered eligible when experimental manipulations aimed to elicit adverse responses through contextual or informational cues, even when formal conditioning paradigms were not explicitly implemented.

Experimental manipulations included both procedures intended to elicit adverse symptom changes and vignette-based or informational manipulations designed to influence the interpretation or attribution of symptoms, provided these were framed within a nocebo context.

Studies were excluded if they did not include an experimental manipulation, involved healthy participants only, or examined adverse outcomes without an explicit attempt to experimentally induce adverse responses or symptom interpretations.

### 2.3 Information sources and search strategy

A systematic literature search was conducted on 23 April 2023 using the databases MEDLINE, PsycINFO, Embase, CINAHL, and Web of Science. Searches were limited to peer-reviewed journal articles published in English.

The search strategy prioritised the use of explicit nocebo-related terminology to maintain conceptual specificity and reproducibility. While it is acknowledged that experimentally induced adverse outcomes may also be indexed under broader constructs such as expectancy, verbal suggestion, or conditioning, these terms were not included as primary search keywords in order to avoid the inclusion of studies that did not explicitly conceptualise adverse outcomes within a nocebo framework. This approach may have limited the sensitivity of the search and is considered in the limitations.

Full database-specific search strategies, including keywords and syntax, are reported in **Supplementary Material A**.

### 2.4 Study selection

All records identified through the database searches were imported into reference management software and duplicates were removed. Titles and abstracts were screened against the eligibility criteria, followed by full-text screening of potentially relevant articles. Screening was conducted to identify experimental studies that aimed to induce adverse outcomes via contextual or informational manipulations in individuals with long-term conditions, including vignette-based paradigms targeting symptom interpretation.

During screening, studies were not excluded on the basis of whether adverse outcomes were induced via expectancy-based or learning-based mechanisms, provided that the experimental manipulation was intended to elicit adverse responses or interpretations through contextual or informational cues.

**Figure 1:**
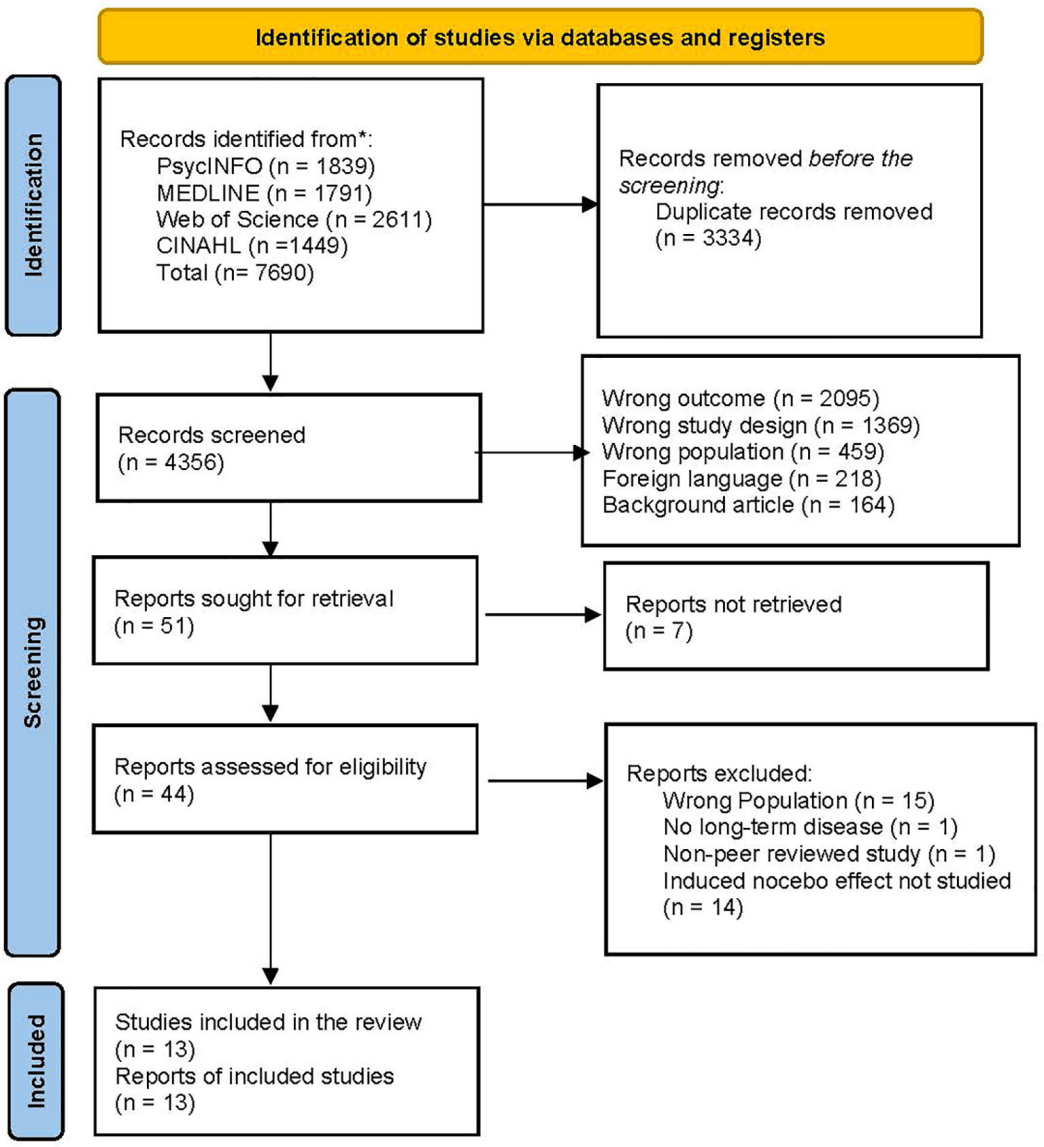
PRISMA flow diagram. Flow of records through the systematic review process, including database searching, screening, eligibility assessment, and final inclusion.

The study selection process is summarised in a PRISMA flow diagram (**Figure 1**).

### 2.5 Data extraction

Data were extracted using a standardised extraction form. Extracted information included study characteristics, such as sample size and health condition; experimental design features; nocebo induction methods; outcome measures; and information on psychological variables or moderators where assessed. Where reported, mean scores, standard deviations, and other descriptive statistics for nocebo-related outcomes were extracted for both experimental and control conditions.

### 2.6 Quality appraisal

Methodological quality of included studies was assessed using the Mixed Methods Appraisal Tool (MMAT) (23). Quality appraisal was conducted to inform interpretation of findings rather than to exclude studies based on methodological quality alone. The MMAT was developed to enable the appraisal of heterogeneous evidence within systematic reviews that include qualitative, quantitative, and mixed-methods designs. Unlike design-specific tools that require separate instruments for randomised trials, non-randomised studies, and descriptive research, the MMAT provides a unified framework.

### 2.7 Data synthesis

Given the substantial heterogeneity across studies in terms of experimental design, nocebo induction methods, outcome measures, and health conditions examined, a meta-analysis was not conducted. Instead, findings were synthesised narratively, with studies grouped according to health condition and outcome type, for example symptom change versus symptom attribution.

## 3 Results

A full flow chart of the extraction is shown through a PRISMA diagram, shown in **Figure 1**.

### 3.1 Study characteristics

Thirteen studies met inclusion criteria and investigated experimentally induced nocebo effects in individuals with long-term health conditions (24–36). These studies are summarised in **Table 1**. For a more detailed extraction, please see **Supplementary Table 1**.

**Table 1:** Summary table.

Study	Country	LTC population	N analysed	Mean age (SD)	% Female	Design	Outcome type
Atmaca et al., 2018 (24)	Turkey	Psychiatric outpatients receiving psychotropic medication	116	38.12 (11.88)*	74%	Non-randomised parallel-group	Clinical side-effect reporting
Barth et al., 2021 (25)	Switzerland	Adults with chronic low back pain	152	39.54 (12.52)	65.80%	Randomised, single-blind 2 × 2 factorial clinical trial	Clinical pain and side-effect reporting
Ciaramella et al., 2013 (26)	Italy	Adults with chronic pain (mixed aetiology)	86	Not reported	Not reported	Single-group experimental study	Experimentally induced symptoms (pain) + clinical pain follow-up
Colagiuri et al., 2013 (27)	Australia	Adults receiving first-time chemotherapy for cancer	91	60.5 (12.4)	57%	Randomised between-subjects	Clinical side-effect reporting
Gröschel et al., 2025 (28)	Netherlands	Women with breast cancer undergoing first-time AC chemotherapy	27	48.4 (11.5)	100%	Pilot 2 × 2 randomised factorial trial	Clinical side-effect reporting + psychological outcomes
Heller et al., 2015 (29)	United States	Adults with and without self-reported asthma (vignette medication scenario)	690	36.2 (12.8)	65.70%	Randomised analogue online experiment	Symptom attribution (analogue)
Jaén & Dalton, 2014 (30)	United States	Adults with moderate persistent asthma	17	38.5 (12.5)*	47%	Between-subject laboratory experiment	Disease-intrinsic symptoms (subjective + physiological)
Keitel et al., 2013a (31)	Germany	Tremor-dominant Parkinson’s disease with chronic STN-DBS	24	64.2 (≈7.8)*	21%	Within-subject repeated-measures	Disease-intrinsic motor symptoms
Keitel et al., 2013b (32)	Germany	Hypokinetic–rigid Parkinson’s disease with chronic STN-DBS	24	62.8 (≈9.3)*	50%	Within-subject repeated-measures	Disease-intrinsic motor symptoms
Malfliet et al., 2018 (33)	Spain	Adults with chronic mechanical neck pain	83	36.6 (≈9.4)*	61%	Three-arm randomised clinical trial	Disease-intrinsic pain symptoms
Napadow et al., 2015 (34)	United States	Adults with atopic dermatitis (chronic itch)	14	25.4 (9.1)	57%	Within-subject experimental fMRI study	Experimentally induced symptoms (itch) + neuroimaging
Sertkaya & Özkaya, 2019 (35)	Turkey	Men with benign prostatic hyperplasia initiating silodosin	120	63.2 (4.4)	0%	Randomised parallel-group	Clinical side-effect reporting
Vase et al., 2003 (36)	United States	Women with irritable bowel syndrome (IBS)	13	30.0 (13.0)	100%	Within-subject experimental study (5 conditions)	Experimentally induced visceral and cutaneous pain

*Standard Deviation has been calculated by B.C based on available data.

Conditions studied included chronic pain (25, 26, 33), Parkinson’s disease (31, 32), irritable bowel syndrome (36), asthma (30), atopic dermatitis (34), benign prostatic hyperplasia (35), chemotherapy-treated cancer populations (27, 28), and psychiatric outpatient samples receiving psychotropic medication (24). One study used an analogue medication paradigm in adults with chronic conditions (29).

Study designs were predominantly experimental. Seven studies employed within-subject paradigms (30–32, 34, 36), four used between-subject or parallel-group designs (24, 25, 33, 35), and two embedded expectancy manipulations within clinical treatment contexts (27, 28).

Nocebo induction methods were primarily verbal and expectancy-based, including negatively framed treatment information (24, 35), explicit suggestion of symptom worsening (31, 32, 36), framing of inert stimuli as potentially harmful (30, 34), and expectancy assessment paradigms (27). Conditioning or contextual expectancy elements were less frequently employed (34, 36).

Outcome measures were predominantly subjective symptom ratings, including visual analogue scales and numerical rating scales (25, 26, 33, 34, 36). Several studies incorporated condition-specific instruments (24, 27, 35). Physiological or objective indices were measured in a minority of studies, including fractional exhaled nitric oxide (30), salivary cortisol (33), and functional neuroimaging (34).

Statistical approaches varied and included repeated-measures ANOVA (30–32, 36), regression analyses (26, 27, 29), logistic regression (27), non-parametric tests (25), and descriptive reporting (28) Standardised effect sizes were inconsistently reported as shown in **Table 2**.

**Table 2:** Nocebo outcomes and moderator effects.

Study	Primary nocebo outcome	Significant nocebo effect?	Magnitude reported	Statistical approach	Significant moderators
Atmaca et al., 2018 (24)	Side-effect reporting (UKU); medication discontinuation	Yes (uninformed group reported more side effects and higher discontinuation)	No standardised effect sizes reported; p-values only	t-tests; χ²; ANCOVA	None
Barth et al., 2021 (25)	Adverse side-effect reporting during acupuncture; pain intensity	No (intense side-effect briefing increased reporting descriptively but not significantly)	Ratio = 1.31 (95% CI 0.94–1.82); no standardised effect size	ANCOVA; longitudinal zero-inflated negative binomial regression	None
Ciaramella et al., 2013 (26)	Sham-induced pain increase during experimental stimulation	Yes (43% classified as nocebo responders; pain increased significantly across stimulation steps)	Repeated-measures ANOVA: F = 9.81, p <.0001; Regression model R² = 0.60 (clinical outcome model); no standardised effect size reported	Repeated-measures ANOVA; ANCOVA; multiple regression	Psychological variables (hypochondriasis, somatosensory amplification) significantly associated; gender moderated some associations
Colagiuri et al., 2013 (27)	Chemotherapy-related side-effect occurrence and severity	Partially (expectancy assessment did not significantly increase side effects overall; however, higher pretreatment expectancies predicted worse nausea, appetite loss, and sadness)	Nausea occurrence OR = 3.19 (p = .06); Within-group regressions: ORs up to 9.56; b coefficients reported; no standardised effect sizes	Logistic regression; linear regression (covariate-adjusted for age, gender, baseline symptoms)	None tested via interaction; expectancy significantly predicted outcomes
Gröschel et al., 2025 (28)	Chemotherapy-related side-effect expectations and experiences; state anxiety	No clear effect (descriptive comparisons only; no inferential testing conducted)	None reported (descriptive means only)	Planned linear mixed models; analyses not conducted due to small sample; descriptive statistics only	Not assessed
Heller et al., 2015 (29)	Symptom misattribution to fictitious medication; intention to discontinue	Yes (misattribution predicted discontinuation; medication beliefs predicted both outcomes)	Misattribution → discontinuation OR = 8.02 (95% CI 4.69–10.69); multivariable ORs up to 3.07; no standardised effect sizes	Logistic regression (univariate and hierarchical multivariable models)	Medication beliefs significantly predicted outcomes; age inversely associated; no interaction effects with asthma diagnosis or leaflet variation
Jaén & Dalton, 2014 (30)	Airway inflammation (fractional exhaled nitric oxide (FeNO)); asthma symptom ratings	Yes (FeNO significantly increased in asthmogenic framing condition; subjective symptoms showed non-significant trends)	FeNO: F(1,13) = 9.93, p <.007; no standardised effect size reported	ANOVA; repeated-measures ANOVA	
Keitel et al., 2013a (31)	Resting tremor and bradykinesia under nocebo suggestion	No (no significant group-level effects; responder subgroup identified)	Descriptive tremor worsening in responders (mean +39% MedON; +95% MedOFF); no standardised effect sizes	Repeated-measures ANOVA; Friedman tests; non-parametric tests	None (no variables significantly distinguished responders)
Keitel et al., 2013b (32)	Proximal motor performance (diadochokinesia) under nocebo suggestion	Yes (expectation significantly affected proximal motor performance MedOFF; placebo > nocebo)	Repeated-measures ANOVA: F(2,46) = 5.24, p <.01; no standardised effect size reported	Repeated-measures ANOVA; paired t-tests; correlation analysis	Disease duration inversely correlated with placebo-induced improvement; no psychological moderators significant
Malfliet et al., 2018 (33)	Pain intensity (VAS) and pressure pain thresholds under negative expectation	Yes (negative expectation group showed no pain improvement; significant group × time interaction)	Repeated-measures ANOVA: group × time p <.001; no standardised effect size reported	Repeated-measures ANOVA	None (no significant correlations between cortisol change and pain/disability)
Napadow et al., 2015 (34)	Itch intensity (NRS); brain activation (dlPFC, caudate, iPS) under nocebo condition	Yes (nocebo condition significantly increased itch ratings and associated brain activation)	Repeated-measures ANOVA: p <.05; correlation between expectancy and itch intensity r = .63, p <.05; no standardised effect size reported	Repeated-measures ANOVA; correlation analysis; fMRI GLM	Expectancy significantly correlated with itch intensity; gender not significant
Sertkaya & Özkaya, 2019 (35)	Sexual side-effect incidence (reduced semen volume) following silodosin	Yes (side-effect reporting significantly higher in informed group)	Reduced semen volume: 43.3% (informed) vs 13.3% (uninformed), p <.001; no standardised effect size reported	χ² test; independent t-tests	None reported
Vase et al., 2003 (36)	Visceral pain intensity and unpleasantness under nocebo suggestion	Yes (nocebo condition significantly increased visceral pain ratings)	Repeated-measures ANOVA: significant condition effects (p <.05); η² values reported for several condition effects (moderate–large range)	Repeated-measures ANOVA; *post hoc* comparisons	Not assessed

### 3.2 Methodological quality

**Figure 2:**
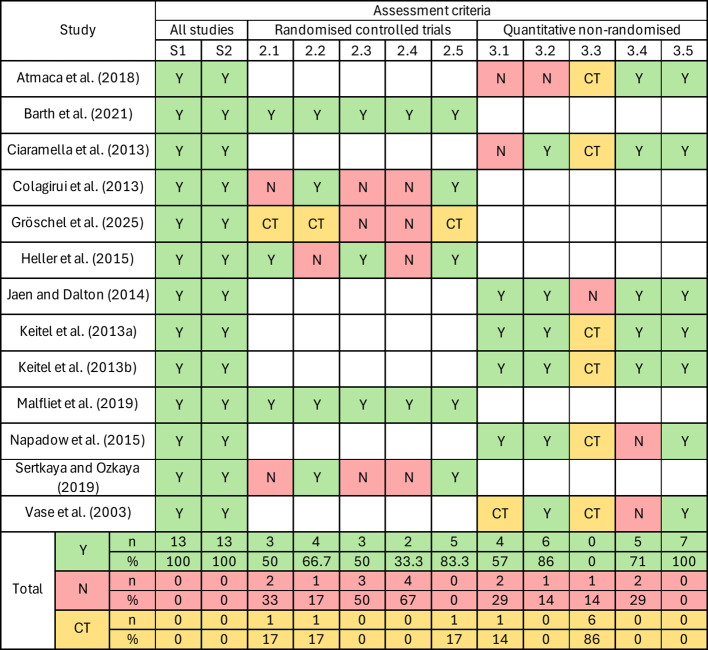
MMAT appraisal of the 13 included studies. Each cell displays the appraisal outcome for a specific MMAT criterion (*Yes*, *No*, or *CT* for “Can’t tell”). S1 = Are there clear research questions?; S2 = Do the collected data allow addressing the research question?; 2.1 = Is randomisation appropriately performed?; 2.2 = Are groups comparable at baseline?; 2.3 = Are there complete outcome data?; 2.4 = Are outcome assessors blinded to the intervention?; 2.5 = Did participants adhere to the assigned intervention?; 3.1 = Are participants representative of the target population?; 3.2 = Are measures appropriate for both outcome and intervention (or exposure)?; 3.3 = Are there complete outcome data?; 3.4 = Are confounders accounted for in the design and analysis?; 3.5 = Was the intervention administered (or exposure occurred) as intended?

Methodological quality was assessed using the Mixed Methods Appraisal Tool (MMAT), with ratings summarised in **Figure 2**.

Overall, most studies demonstrated clear research questions and appropriate alignment between research design and study objectives. Experimental manipulations were generally well-defined, and analytic approaches were appropriate for the respective study designs.

However, several recurring methodological limitations were identified. Sample sizes were small in a number of laboratory-based studies, particularly within-subject experimental paradigms. Reporting of effect sizes was inconsistent across studies. In some cases, representativeness of the recruited samples was unclear, and allocation procedures or blinding were not fully described in between-group designs.

Attrition and adherence were variably reported, particularly in studies embedded within clinical treatment contexts. Additionally, expectancy-based paradigms inherently limited participant blinding, which may have introduced performance bias.

Despite these limitations, most studies demonstrated internal coherence between experimental manipulation, outcome measurement, and statistical analysis. No study was excluded on the basis of methodological quality.

### 3.3 Nocebo effects on treatment-related side-effect incidence

Five studies examined nocebo effects in relation to treatment-related side-effect reporting (24, 25, 27, 29, 35).

In psychiatric outpatients, provision of detailed side-effect information was associated with altered patterns of reported adverse effects and differences in medication discontinuation rates (24). In benign prostatic hyperplasia, informing patients about potential sexual adverse effects significantly increased reporting of reduced semen volume (35). In chemotherapy-treated patients, baseline side-effect expectancy predicted subsequent nausea and related symptoms after adjustment for baseline symptom levels; however, expectancy assessment alone did not significantly increase overall side-effect incidence (27). In chronic low back pain, intensified adverse-effect framing during acupuncture did not significantly increase adverse-event reporting (25). In an analogue medication paradigm, symptom misattribution to a fictitious medication occurred in approximately one quarter of participants and was strongly associated with intended discontinuation, with medication beliefs emerging as significant predictors (29).

Across these studies, nocebo effects were primarily expressed as increased symptom reporting or attribution rather than experimentally induced physiological deterioration. Statistical analyses included logistic regression, ANCOVA, and group comparisons. Effect sizes were reported as odds ratios or regression coefficients in some studies (27, 29), whereas others reported p-values without standardised magnitude indices (24, 25, 35).

### 3.4 Nocebo effects on disease-specific symptoms

Four studies examined nocebo effects on symptoms intrinsic to participants’ underlying long-term conditions (30–33).

In Parkinson’s disease, negative expectancy significantly reduced proximal motor performance under medication withdrawal in hypokinetic–rigid presentations, whereas group-level effects were not consistently observed in tremor-dominant samples (31, 32). In asthma, framing an inert odour as harmful significantly increased airway inflammation measured via exhaled nitric oxide, although spirometric indices were not significantly altered (30). In chronic mechanical neck pain, negative treatment expectations attenuated improvements in pain intensity and were associated with increased salivary cortisol, while disability and range of motion were not significantly altered (33).

Studies predominantly employed within-subject or repeated-measures designs. Statistical significance was reported across conditions; however, standardised effect size reporting was inconsistent (30–33). Effects were most consistently observed in subjective symptom ratings or motor performance indices, with physiological measures demonstrating more selective modulation.

### 3.5 Nocebo effects on experimentally induced symptoms

Three studies investigated nocebo effects on experimentally induced symptoms independent of spontaneous disease processes (26, 34, 36).

In chronic pain, sham electrical stimulation paired with suggestion of increasing intensity elicited nocebo responses in approximately 43% of participants, with psychological variables such as somatosensory amplification and distress associated with greater response magnitude (26). In atopic dermatitis, saline presented as an allergen significantly increased itch ratings relative to open control conditions and was associated with increased activation in prefrontal and striatal brain regions (34). In irritable bowel syndrome, negative verbal suggestion during rectal distension significantly increased visceral pain ratings relative to control conditions, and expectancy-related variables accounted for substantial proportions of variance in pain ratings (36).

Statistical methods varied across studies. Regression-based R² values were reported for psychological predictors in chronic pain (26), partial η² values were reported for repeated-measures effects in visceral pain paradigms (36), and correlational analyses were reported in neuroimaging contexts (34). Across paradigms, nocebo effects were most robust in subjective symptom ratings, with physiological and neurobiological measures showing selective and context-dependent modulation.

### 3.6 Magnitude of reported effects

Standardised effect size reporting was inconsistent across studies. Partial η² values were reported for condition effects in experimentally induced visceral pain paradigms, with several effects falling in the moderate to large range (36). Regression-based R² values were reported for psychological predictors of nocebo magnitude in chronic pain (26). Odds ratios and regression coefficients were reported in models of symptom attribution and side-effect expectancy (27, 29). In contrast, several studies reported statistical significance without accompanying standardised magnitude indices (24, 25, 30–33, 35). One pilot study reported descriptive findings without inferential testing (28). Consequently, although moderate to large effects were observed in some experimental paradigms, cross-study comparison of effect magnitude was limited. **Table 2** summarises the primary nocebo outcomes reported in each study, including whether statistically significant effects were observed, the statistical approach used, reported inferential statistics, and any effect size indices provided by authors.

### 3.7 Moderators of nocebo effects

Eight studies examined potential moderators of nocebo responses (24–26, 29, 31–34).

Demographic variables such as age and sex were generally not significant moderators (24, 34, 35). In Parkinson’s disease, disease duration was associated with expectancy-related motor effects in one study, although findings were not consistent across subtypes (31, 32). Psychological variables were more frequently associated with nocebo magnitude. Medication beliefs predicted symptom misattribution and behavioural intention (29). Somatosensory amplification, hypochondriasis, and distress were associated with stronger nocebo responses in chronic pain (26). Expectancy ratings were positively correlated with nocebo-induced itch intensity (34). Anxiety was measured in multiple studies, but associations with nocebo magnitude were inconsistent (25, 28, 33).

### 3.8 Coverage of long-term conditions and gaps

Evidence was concentrated in pain-related and neurological conditions (25, 26, 31–33). Gastrointestinal and dermatological conditions were represented by irritable bowel syndrome and atopic dermatitis (34, 36), while respiratory and urological conditions were represented by asthma and benign prostatic hyperplasia (30, 35). Oncological and psychiatric medication contexts were also included (24, 27, 28).

Cardiovascular, metabolic, autoimmune, and multimorbid long-term conditions were not represented in the included experimental studies. Experimentally induced nocebo research in long-term conditions therefore remains concentrated in a limited subset of disease domains, with substantial gaps across other chronic illness populations.

## 4 Discussion

This systematic review synthesised experimental evidence examining nocebo effects in long-term conditions. Across heterogeneous clinical populations and paradigms, negative expectations were capable of influencing symptom reporting, behavioural intention, and in some cases physiological indices. However, effects were neither universal nor consistent across outcome domains. The current evidence base supports the plausibility of nocebo mechanisms in chronic illness, while also highlighting important contextual and methodological constraints.

Across medication-based and information-based paradigms, expectancy and belief systems emerged as particularly influential. In analogue and clinical designs, medication-related beliefs robustly predicted symptom misattribution and behavioural intention independent of framing manipulations (29). Informing patients about potential sexual side effects increased reporting of reduced semen volume in men initiating silodosin (35). In contrast, detailed side-effect information in psychiatric outpatients was associated with lower overall side-effect burden and reduced discontinuation rates (24). These findings indicate that information does not uniformly increase harm; rather, its impact appears to depend on context, reassurance, prior beliefs, and interpretive frameworks. This pattern aligns with expectancy-based models in which prior beliefs and threat appraisal shape symptom perception and attribution (5, 8).

Experimental induction paradigms further demonstrated that negative expectations can modulate both subjective and physiological outcomes. In asthma, framing an inert odor as harmful significantly increased airway inflammation measured via exhaled nitric oxide, despite no significant changes in spirometry (30). In chronic neck pain, negative treatment expectations attenuated pain improvement relative to positive and neutral expectations, although disability outcomes were unaffected (33). In atopic dermatitis, saline presented as an allergen increased itch ratings and activated prefrontal and striatal regions implicated in expectancy processing (34). In irritable bowel syndrome, expectancy accounted for a substantial proportion of variance in experimentally induced visceral pain (36). Together, these findings support the view that negative expectations can influence perceptual and neurophysiological processes relevant to chronic symptom experience.

However, nocebo effects were not consistently observed across all conditions. In chronic low back pain, intensified adverse side-effect briefing did not significantly increase adverse-effect reporting or pain intensity (25). In tremor-dominant Parkinson’s disease, no significant group-level nocebo effects were observed, although exploratory responder analyses suggested individual variability (31, 32). A pilot feasibility trial examining nocebo-alleviating communication and clinician-expressed empathy in breast cancer patients reported no clear inferential effects on side-effect outcomes (28). These null or mixed findings underscore the context-dependent nature of nocebo phenomena and suggest that expectancy effects interact with disease characteristics, study design, and individual psychological profiles.

A consistent pattern across the included studies was the greater robustness of effects on subjective symptom ratings compared with objective physiological indices. While inflammatory modulation was observed in asthma (30) and neural activation changes were demonstrated in itch paradigms (34), many objective measures were unaffected even when subjective outcomes changed (31–33). This dissociation is consistent with predictive processing and expectancy-based accounts, in which cognitive-affective mechanisms influence perception, interpretation, and reporting of bodily signals, with more variable downstream physiological consequences (8, 37).

Psychological variables were more consistently associated with nocebo responses than demographic characteristics. Somatosensory amplification, hypochondriasis, and related constructs were associated with nocebo response magnitude in chronic pain (26), and medication beliefs robustly predicted both symptom misattribution and intention to discontinue treatment (29). By contrast, age and gender were generally weak or inconsistent predictors. Nevertheless, formal moderation analyses were not consistently implemented, and many studies relied on covariate adjustment rather than interaction testing. Consequently, mechanistic inference remains limited.

## 5 Strengths and Limitations

A strength of this review is the integration of laboratory-based expectancy paradigms with clinically embedded studies across multiple long-term conditions. Detailed extraction of statistical approaches and moderator analyses enhances transparency regarding evidential strength. The inclusion of both subjective and physiological outcomes allows consideration of mechanistic breadth.

Limitations include heterogeneity of design and outcome measurement, inconsistent reporting of standardised effect sizes, and generally modest sample sizes. The absence of meta-analysis limits quantitative precision. Additionally, many studies examined short-term or experimentally induced outcomes rather than long-term disease trajectories, constraining inference regarding sustained clinical impact.

Methodologically, several features constrain interpretation. Standardised effect sizes were infrequently reported, despite statistically significant findings in multiple studies, with most analyses relying on p-values, F statistics, or regression coefficients without accompanying standardised indices (24–26, 30–32, 35). This limits cross-study comparison and precludes quantitative synthesis. Sample sizes were often modest, particularly in laboratory-based paradigms, increasing vulnerability to both Type I and Type II error. Designs varied considerably, including analogue online studies, laboratory induction paradigms, and clinical trials embedded within treatment contexts. While this enhances ecological breadth, it reduces methodological uniformity.

The search strategy was intentionally anchored to the conceptual construct of the nocebo effect rather than to a broad array of loosely related expectancy terms. This approach prioritised conceptual specificity and theoretical coherence. However, variability in terminology across clinical and experimental literatures means that some studies examining negative expectancy effects without explicit nocebo framing may not have been retrieved. Terminological inconsistency remains a structural challenge in this field.

## 6 Conclusion

Evidence across long-term conditions indicates that negative expectations can influence symptom perception, reporting, and behavioural intention, and may in some contexts modulate physiological processes. However, effects are context-dependent, outcome-specific, and variably moderated by psychological factors. Future research should prioritise adequately powered designs, consistent effect size reporting, and formal testing of mechanistic pathways. A theoretically grounded and methodologically rigorous approach will be essential to clarify when and how nocebo effects meaningfully influence chronic disease management.

## Data Availability

The original contributions presented in the study are included in the article/**Supplementary Material**. Further inquiries can be directed to the corresponding author.
