# Supplementary material for: Nocebo effects in long-term health conditions: a systematic review of experimental studies
Source: Front Psychiatry. 2026 May 8;17:1752434. doi: 10.3389/fpsyt.2026.1752434 (PMC13194134; doi:10.3389/fpsyt.2026.1752434)
Supplement: Supplementary file 1 [file Table1.docx]

Supplementary File A

On 23 April 2023, a literature search was conducted using the keywords “nocebo,” “negative placebo,” “placebo side effect$,” “psychogenic symptom$,” “sociogenic symptom$,” “psychogenic illness,” and “sociogenic illness.”
