# Supplementary material for: Nocebo effects in long-term health conditions: a systematic review of experimental studies
Source: Front Psychiatry. 2026 May 8;17:1752434. doi: 10.3389/fpsyt.2026.1752434 (PMC13194134; doi:10.3389/fpsyt.2026.1752434)
Supplement: Supplementary file 2 [file Table2.docx]

Supplementary File B

**Title and Abstract Screening Tool**

- Has the study been published in a peer-reviewed journal?
- Has the study reported findings in English?
- Has the study been reported as a review or meta-analysis?
- Has the study used animals?
- Has the study exclusively used a qualitative approach?
- Has the study used an experimental design?
- Has the study included at least one arm diagnosed with a long-term condition?
- Has the study reported the effect size?
- Has the study induced the nocebo effect? (not an observational study)
