# Supplementary material for: Nocebo effects in long-term health conditions: a systematic review of experimental studies
Source: Front Psychiatry. 2026 May 8;17:1752434. doi: 10.3389/fpsyt.2026.1752434 (PMC13194134; doi:10.3389/fpsyt.2026.1752434)
Supplement: Supplementary file 3 [file Table3.docx]

Supplementary File C

MMAT Questions

S1. Are there clear research questions?

S2. Do the collected data allow to address the research question?

**RCT**

2.1 Is randomisation appropriately performed?

2.2 Are the groups comparable at baseline?

2.3 Are there complete outcome data?

2.4 Are outcome assessors blinded to the intervention provided?

2.5 Did the participants adhere to the assigned intervention?

**Non-Randomised Quantitative**

3.1 Are the participants representative of the target population?

3.2 Are measures appropriate regarding both the outcome and intervention (or exposure)?

3.3 Are there complete outcome data?

3.4 Are the confounders accounted for in the design and analysis?

3.5 During the study period, is the intervention administered (or exposure occurred) as intended?
